# Risk and Predictive Factors of Leptospirosis in Dogs Diagnosed with Kidney and/or Liver Disease in Selangor, Malaysia

**DOI:** 10.3390/ani11123405

**Published:** 2021-11-29

**Authors:** Mohammad Sabri Abdul Rahman, Kuan Hua Khor, Siti Khairani-Bejo, Seng Fong Lau, Mazlina Mazlan, Mohd Azri Roslan

**Affiliations:** 1 Department of Veterinary Clinical Studies, Faculty of Veterinary Medicine, Universiti Putra Malaysia, Serdang 43400, Selangor, Malaysia; sabrahman90@gmail.com (M.S.A.R.); lausengfong@upm.edu.my (S.F.L.); 2 Department of Veterinary Pathology and Microbiology, Faculty of Veterinary Medicine, Universiti Putra Malaysia, Serdang 43400, Selangor, Malaysia; skhairani@upm.edu.my (S.K.-B.); m_mazlina@upm.edu.my (M.M.); m_azri@upm.edu.my (M.A.R.)

**Keywords:** dogs, kidney and/or liver disease, leptospirosis, predictive, risk

## Abstract

**Simple Summary:**

Canine leptospirosis is commonly associated with kidney and/or liver disease. The current study identified the potential risk and predictive factors of dogs diagnosed with kidney and/or liver disease due to leptospirosis. A total of 124 client-owned dogs were recruited and the samples collected were directly tested using polymerase chain reaction (PCR) and subsequently inoculated for bacterial isolation. Statistical analyses were descriptively analyzed, and risk analyses were performed using Pearson chi-square tests and logistic regression. The different breeds of dog with history of exposure to rats and those managed outdoors had a greater risk of leptospirosis (*p* < 0.05). The significant predictors for the dogs’ positivity were presence of rats and acute clinical illness (*p* < 0.05). Administration of antibiotics and detection of clinical illness at an early stage of the disease improved the survivability of the dogs (*p* < 0.05).

**Abstract:**

Canine leptospirosis is commonly associated with kidney and/or liver disease. It has been widely reported and causes public health concerns due to its zoonotic potential and its re-emergence, resulting from close contact between humans and dogs. The current study identified potential risk and predictive factors for dogs diagnosed with kidney and/or liver disease due to leptospirosis. A total of 124 client-owned dogs were recruited, and information such as signalment, medical history, management, and clinical findings were documented. Samples collected from the recruited dogs were directly tested using polymerase chain reaction (PCR) and subsequently inoculated for bacterial isolation. Statistical analyses were descriptively analyzed, and risk analyses were performed using Pearson chi-square tests and logistic regression. A total of 53 dogs (42.7%) were positive for leptospiral infection based on PCR, and 10 leptospiral isolates were successfully recovered from eight dogs. The mortality rate of infected dogs was 34.0% (18/53). Medium and large dog breeds, with a history of exposure to rats, and managed outdoors had a greater risk for leptospirosis (*p* < 0.05). The significant predictors for the dogs’ positivity were the presence of rats and acute clinical illness (*p* < 0.05). Administration of antibiotics and the detection of clinical illness at an early stage of the disease improved the survivability of the dogs (*p* < 0.05). Identifying the profile of dogs that are at risk to leptospirosis could be useful in the design of diagnostic and treatment strategies, as well as to increase awareness for prevention of the disease.

## 1. Introduction

Leptospirosis is a zoonotic bacterial disease with a global distribution. It is caused by spirochetes of the *Leptospira* genus, which are further divided into pathogenic and saprophytic species, with more than 250 pathogenic, and 60 saprophytic, serovars [1]. Its incidence is increasingly recognized in developed, high-income countries, but the highest burden remains in sub-tropical and tropical regions worldwide, especially in developing countries [1]. Disease transmission occurs through direct contact with urine contaminated with leptospires or indirect contact with contaminated moist environments [2]. Goarant (2016) reported that animals mostly become infected through environmental exposure [3].

Reservoirs for leptospirosis can be either wild or domestic animals, such as rodents, cattle, or dogs [4]. Dogs are considered to be highly susceptible, and canine leptospirosis has been widely described worldwide [5,6]. The circulation of *Leptospira* spp. among wildlife is an important transmission route, especially for hunting dogs [7]. Although dogs are considered reservoirs of *Leptospira interrogans* serovar Canicola, canine leptospirosis can also be caused by other serovars [8,9]. Recent reports have reported the re-emergence of clinical illness in both dogs and humans [6,10].

Previous canine risk factors and prevalence studies have used microscopic agglutination test (MAT) data [11,12,13], but this assessment method could not differentiate between vaccination and post-exposure. In recent years, polymerase chain reaction (PCR) has become increasingly adopted to detect *Leptospira* spp., as it may reduce the interpretation challenges commonly encountered with MAT. Results from PCR are not influenced by history of vaccination, and since it does not identify the leptospiral serovars, there are no concerns about cross reaction among serovars [14,15]. Nonetheless, culturing leptospires still stands as the gold standard reference test for confirmation of leptospiral infection, in combination with serological characterization of the isolated strains; with both tests providing reliable information regarding serovar identity [6].

The risk factors for human infection with leptospirosis include occupational, recreational, sportive activities, and ecotourism [16,17]. However, the risk factors for leptospiral infection in pet dogs in Malaysia are unknown, and understanding these risks may aid in clinical decision making. This study identified the important risk factors and predictors of leptospirosis in client-owned dogs diagnosed with kidney and/or liver disease, with the utilization of PCR, isolation, and identification of leptospires as diagnostic methods in Selangor, Malaysia.

## 2. Materials and Methods

### 2.1. Sample Collection and Inclusion Criteria

Client-owned dogs diagnosed with kidney and/or liver disease presented at the University Veterinary Hospital (UVH), Faculty of Veterinary Medicine (FVM), Universiti Putra Malaysia (UPM), or from private veterinary clinics within a 10-km radius from UVH were recruited. Dog owner’s consent was obtained, and the selection criteria of the recruited dogs were (i) dogs presenting with clinical signs of kidney and/or liver disease and (ii) elevated kidney (urea (>7.5 mmol/L), creatinine (>176 µmol/L)) and/or liver (alanine aminotransferase (ALT) (>90 U/L) and alkaline phosphatase (ALP) (>100 U/L)) parameters, based on serum biochemistry profile. The average and standard deviation (SD) values for biochemical laboratory tests were recorded. The average kidney parameters were graded based on published guidelines of kidney injury [18]. The average increases in liver parameters were interpreted as mild (2- to 3-fold elevation), moderate (5- to 10-fold elevation) and severe (>10-fold elevation) hepatocellular injury [19]. The signalment, history, clinical findings, treatment, and survivability of each dog were recorded. Ethical approval for this study was obtained from the Institutional Animal Care and Use Committee (IACUC Ref No: UPM/IACUC/AUP-R084/2016).

Each dog was manually restrained for venipuncture, and urine samples were collected via ultrasound-guided cystocentesis by experienced veterinarians. Blood collected was stored in blood tubes containing the anticoagulant ethylenediaminetetraacetic acid (EDTA) (BD Vacutainer^®^, Franklin Lakes, NJ, USA) and urine samples were collected in sterile universal containers. Both samples were stored and maintained at 4 °C and immediately transferred to the laboratory for further analyses. 

### 2.2. Molecular Detection Using Polymerase Chain Reaction (PCR)

DNA extraction from the samples (whole blood, urine, and positive control of *Leptospira interrogans* serovar Canicola strain Hond Utrecht IV were performed using a DNeasy^®^ Blood & Tissue Kit (QIAGEN, Hilden, Germany), as described in the manufacturer’s protocol. The end products (DNA template) were inspected using 1.5% agarose gel for purity. Two sets of primers were selected and targeted the 16S rRNA and LipL32 genes [20,21]. The forward and reverse primers for 16S rRNA are 5′–CATGCAAGTCAAGCGGAGTA–3′ and 5′–AGTTGAGCCCGCAGTTTTC–3′, respectively, with amplicon size of 541 base pair (bp). The forward and reverse primers for LipL32 are 5′– GTCGACATGAAAAAACTTTCGATTTTG–3′ and 5′–CTGCAGTTACTTAGTCGCGTCAGAAGC–3′, respectively, with amplicon size of 756 bp. 

A total reaction volume of 25.0 μL was optimized as follows: 12.5 μL 2× MyTaq™ Red Mix (BIOLINE, London, UK), 2.5 μL with a concentration of 10 µM for the primer (forward and reverse) and a 10.0 μL DNA template. Amplification was optimized with an initial denaturation of 94 °C for 5 min followed by 30 cycles of denaturation at 94 °C for 1 min, primer annealing at 58 °C for 45 s, and DNA extension at 72 °C for 30 s, before the final extension step at 72 °C for 6 min to complete the synthesis of all strands. The amplicons were analyzed in tris-borate-EDTA (TBE) buffer at 80 volts for 1.5 h by using 1.5% gel electrophoresis. The gel was pre-stained with SYBR^®^ Safe DNA gel stain (Invitrogen™, Waltham, MA, USA) and examined using Gel Documentation (AlphaImager™, Santa Clara, CA, USA). The amplicons were identified by their band sizes. Both genes were present in pathogenic *Leptospira* spp., but only 16S rRNA gene was present in the non-pathogenic *Leptospira* spp. [22].

### 2.3. Isolation and Identification of Leptospira spp. 

The isolation of *Leptospira* spp. in this study was based on the protocol described by the World Organization for Animal Health (OIE) [23]. Two drops of whole blood and urine and were inoculated into semisolid Ellinghausen and McCullough modified by Johnson and Harris (EMJH) medium, which contained 200 µg/mL 5-fluorouracil. The inoculation of all the samples was performed within 2 h of collection from the dogs, and the primary cultures were maintained in an incubator (30 °C) for 12 weeks. The cultures were checked every 2 weeks to check for the presence of leptospires under darkfield microscopy. If leptospires were observed within 12 weeks, the positive cultures were transferred into liquid EMJH medium to enhance their growth and filtered with a 0.45 µM filter (Millex^®^, Dublin, Ireland) until pure isolates were obtained. 

The pure isolates were maintained in liquid EMJH medium and further identified through serotyping using 18 hyperimmune sera, namely Australis, Autumnalis, Ballum, Bataviae, Celledoni, Copenhageni, Cynopteri, Djasiman, Hardjobovis, Hebdomadis, Icterohaemorrhagiae, Javanica, Lai, Malaysia, Patoc, Pomona, Pyrogenes, and Tarassovi. The hyperimmune sera were provided by Forensic and Scientific Services, Department of Health, Leptospirosis Reference Laboratory, Queensland, Australia. Multilocus sequence typing (MLST) of the isolates using 7 distinct loci (pntA, sucA, mreA, glmU, caiB, tpiA, and pfkB) was performed, as previously described [24]. The concatenated loci were compared to *Leptospira* sequence types (STs) available in the PubMLST database (https://pubmlst.org/leptospira/; accessed on 2 April 2021). The cultures were discarded after a final careful examination if found negative for leptospires within the 12-week incubation period. Leptospiral isolation and identification were performed to determine the infecting serovar in this study. 

### 2.4. Risk Factors Analysis

Demographic (age, breed, sex, and vaccination records) and environmental (rat exposure) information of the dogs was recorded. All the responses to the factors were dichotomously recorded, except age and breed (record as trichotomous, based on published guidelines [25]). Additional information, such as type of management, type of household, duration of clinical illness, and antibiotic given prior presentation, was also recorded.

The factors used for analysis were as follows: (i) rat exposure, (ii) management, specifically, indoor/outdoor status, (iii) type of household (specifically, whether or not the dog was in a multiple dog household), (iv) duration of clinical illness, and (v) prior antibiotic administration. Dogs were considered exposed to rats if either the owner reported dog contact with rats (chasing, catching, or eating a rat(s)) or the owner reported the presence of rats or rat feces within the house compound. Dogs were considered to be indoor if they were kept inside the house, and outdoor if they were kept outside of the house. Dogs were considered to live in a multiple-dog household if more than one dog lived in the household. Acute illness was defined as clinical signs of less than or equal to 7 days duration, while chronic illness was defined as a greater than 7 day duration.

### 2.5. Statistical Analysis

The age, breed, sex, vaccination status, type of management, type of household, rat exposure, and duration of clinical illness were defined as potential risk factors. A Pearson chi-square test was performed for each factor to determine the association between risks and leptospiral infection. Logistic regression was also performed to predict the likelihood of leptospiral infection when exposed to the risks (sex, vaccination status, type of management, type of household, rat exposure, and duration of clinical illness). Odds ratios (OR) were estimated with 95% confidence intervals. Mortality rate was calculated based on the dogs that died over positive cases. A Pearson chi-square test on antibiotics given prior to presentation, duration of clinical illness, and vaccination status was also performed to determine the association between these 3 factors and the survival of the dogs. The significance value was determined at a *p*-value less than 0.05 (*p* < 0.05).

## 3. Results

Out of the 124 dogs recruited in this study, 68 dogs were diagnosed with both kidney and liver diseases, whereas the remaining 34 dogs had kidney disease and 22 dogs had liver disease. Laboratory analyses revealed that the majority of the recruited dogs were diagnosed with moderate acute kidney injury and/or mild hepatocellular injury (Table 1).

The demographic data of the dogs are shown in Table 2. The majority of the dogs were in the senior age group, male, non-vaccinated, and medium-sized dogs. Most of these dogs were managed outdoors, in a single dog household, had rat exposure, and presented with acute clinical illness. A few dog owners anecdotally complained that they had seen rat feces surrounding the dog bowl (*n* = 3) or had seen their dogs catching rats (*n* = 5) within the house compound, with one dog in contact with civet cat. Based on the vaccination records obtained, 41.9% of the dogs were vaccinated with a commercial tetravalent vaccine consisting of the serovars Canicola, Grippotyphosa, Icterohaemorrhagiae, and Pomona. Forty-four out of 124 dogs had received antibiotics prior to presentation, namely amoxicillin (*n* = 2), amoxicillin with combination of clavulanic acid (*n* = 4), ampicillin (*n* = 1), penicillin (*n* = 4), ciprofloxacin (*n* = 1), doxycycline (*n* = 22), and enrofloxacin (*n* = 10). Upon hospitalization, all the dogs were started on antibiotics for treatment and supportive therapy for kidney and liver disease.

The breeds of dog recruited in this study consisted of (i) large breeds (*n* = 31) (American Bulldog (*n* = 1), Bullmastiff (*n* = 1), Doberman (*n* = 2), German Shepherd (*n* = 5), Golden Retriever (*n* = 3), Great Dane (*n* = 1), Labrador (*n* = 7), Mastiff (*n* = 1), Pit Bull (*n* = 1), Pit Bull Cross (*n* = 1), Rottweiler (*n* = 5), Shetland Sheepdog (*n* = 1), Siberian Husky (*n* = 2)), (ii) medium breeds (*n* = 65) (American Cocker Spaniel (*n* = 1), Beagle (*n* = 2), Cocker Spaniel (*n* = 1), Dachshund (*n* = 1), Local Breeds (*n* = 56), Schnauzer (*n* = 1), Schnauzer Mix (*n* = 2), Springer Spaniel (*n* = 1)), and (iii) small breeds (*n* = 28) (Chi Hua Hua (*n* = 2), Jack Russell Terrier (*n* = 1), Maltese (*n* = 3), Mini Pinscher (*n* = 1), Pomeranian (*n* = 4), Poodle (*n* = 6), Shih Tzu (*n* = 9), Silky Terrier (*n* = 1), Terrier Mix (*n* = 1)).

**Figure 1:**
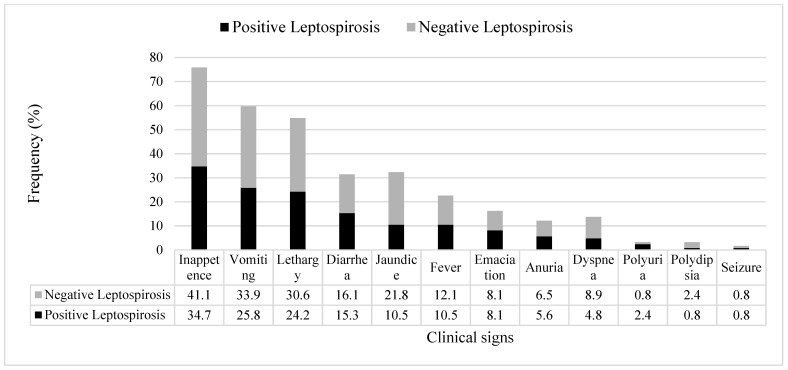
Frequency (%) of clinical signs observed in the dogs diagnosed with kidney and/or liver disease with leptospirosis (black bar) and without leptospirosis (grey bar) that had presented to hospital or private clinics (*n* = 124).

All dogs with kidney and/or liver disease with or without leptospirosis (confirmed by PCR) were observed to have at least two clinical signs, and the most commons signs were inappetence, followed by vomiting, lethargy, jaundice, and diarrhea (Figure 1).

### 3.1. Molecular Detection Using Polymerase Chain Reaction (PCR)

A total of 124 whole blood and 113 urine samples were collected. The incidence of dogs with kidney and/or liver disease and positive for leptospirosis was 42.7% (53/124; 95% CI: 34.0–51.4%), detected from 42 whole blood and 36 urine samples. All these samples were identified positive for pathogenic *Leptospira* spp. Among the 53 infected dogs, 29 dogs presented with both kidney and liver disease, 14 dogs presented with kidney disease only, and another 10 dogs presented with liver disease only. In addition, among these 53 dogs, pathogenic leptospiral DNA was detected in both whole blood and urine in 25 dogs; only whole blood in 17 dogs; and only urine in 11 dogs.

### 3.2. Isolation and Identification of Leptospira spp.

*Leptospira* spp. were successfully isolated from 8 out of 124 dogs (6.5%; 95%CI: 2.1–10.8%). Interestingly, three out of the eight dogs with positive isolates had received vaccination annually. From these eight dogs, 10 isolates were obtained from blood (*n* = 3) and urine (*n* = 7) samples. Serotyping revealed that the majority of the isolates were Bataviae (*n* = 7), followed by Australis (*n* = 2) and Javanica (*n* = 1). MLST analysis of the isolates revealed ST 50 (*n* = 7; *L. interrogans* serogroup Bataviae), ST 51 (*n* = 2; *L. interrogans* serogroup Australis), and ST 143 (*n* = 1; *L. borgpetersenii* serogroup Javanica) (Table 3). 

### 3.3. Statistical Analysis

Both the medium and large breed dogs were 4.19 and 5.59 times more likely (*p* < 0.05) to be infected with *Leptospira* compared to small breed dogs. Dogs that were exposed to rats were at greater risk; 3.14 times more likely (*p* < 0.05) to be infected with *Leptospira* compared to dogs that were not exposed to rats (Table 4). 

As for the breed of dog, the majority of the infected dogs presented were medium breed dogs (*n* = 31), followed by 17 large and five small breed dogs. In the group of dogs that were positive for leptospirosis, information obtained from their owners revealed that most of the dogs (*n* = 32 dogs; 9 large, 21 medium, and 2 small breeds) were kept outdoors and 41 dogs (12 large, 26 medium and 3 small breeds) were exposed to rats. Table 5 shows that dogs-maintained outdoors were 4.31 times more likely to be exposed to rats compared to dogs managed indoors.

Logistic regression was performed to ascertain the effects of rat exposure and the duration of clinical illness on the likelihood that dogs were positive, with sex, vaccination status, type of management, and type of household as controlled factors (Table 6). The model was statistically significant χ^2^ = 12.67, *p* = 0.002. The model explained 13.0% of the variance in the leptospiral positivity and correctly classified 63.7% of cases. Dogs with a history of rat contact were 3.52 times more likely to be positive than those without. Dogs with an acute clinical illness were 2.76 times more likely to be leptospirosis positive than dog with chronic clinical illness.

Out of 53 positive dogs, 18 dogs did not survive: 12 dogs were euthanized when not responding to treatment, and another 6 dogs died due to the sequelae of the disease, with a mortality rate of 34.0% (18/53; 95% CI: 21.2–46.7%). From the history obtained, the antibiotics administered prior to presentation in these infected dogs were ampicillin (*n* = 1), ciprofloxacin (*n* = 1), doxycycline (*n* = 12), enrofloxacin (*n* = 4), and penicillin (*n* = 2). Table 7 shows the association between an antibiotic given prior to presentation (20 dogs given; 33 dogs not given), duration of clinical illness (13 dogs with chronic illness; 40 dogs with acute illness), and vaccination status (21 dogs vaccinated; 32 dogs non-vaccinated) with the survival of the dogs using a Pearson chi-square analysis. There was a statistical significance (*p* < 0.05) between antibiotics, duration of clinical illness, and survival of the dogs. The dogs that were given antibiotics and presented with chronic clinical illness had a higher chance of survival, with an OR of 4.72 and 8.87, respectively. However, there was no significant association (*p* > 0.05) between vaccination and the survival of the dogs.

## 4. Discussion

In Malaysia, most studies of canine leptospirosis have focused on incidence, risk factors, and seroprevalence [10,26,27,28,29]. One study looked at the predictors for canine leptospirosis [10], but focused on different groups of dogs (dogs from working institutions and shelters) and the diagnostic assessment used was MAT. The current study determined the risk and predictive factors for leptospirosis using PCR, for diagnosis in dogs diagnosed with kidney and/or liver disease.

Infected dogs may manifest a broad spectrum of clinical signs and often experience kidney or liver failure [30]. The most common clinical signs associated with canine leptospirosis is kidney injury [31], while vomiting (*n* = 32), lethargy (*n* = 30), polyuria (*n* = 3), and polydipsia (*n* = 1) were the most commonly observed clinical signs in this study. The suspicion of leptospirosis was increased in dogs with kidney injury that had concurrent evidence of hepatocellular injury or cholestatic liver disease, which was manifested as increased serum ALP [32]. Consistent with the findings in this study, 29 dogs infected with leptospirosis had both kidney and liver disease. When hepatitis occurred without kidney injury, it was observed that elevations in serum ALT and ALP occurred, with variable severity [32]. Consistent with the findings in this study, 10 dogs infected with leptospirosis presented with liver disease only (average ALT 189 U/L; average ALP 769.9 U/L; moderate hepatocellular injury). In brief, canine leptospirosis can occur in dogs with kidney and/or liver disease and manifests a wide variety of blood profiles.

The percentage of dogs diagnosed with kidney and/or liver disease caused by leptospirosis was 42.7% (53 dogs) based on molecular testing using PCR. The findings were consistent with a previous study reported at 42.4% (14/33; 95%CI: 25.6–59.3%) [33], despite having a smaller sample size, but with a similar target population. In contrast, Latosinski et al. (2018) [34] and Santanna et al. (2017) [35] reported lower molecular detection rates, at 19.8% (26/131; 95%CI: 13.0–26.7%) and 1.0% (1/106; 95%CI: 0.0–2.8%), respectively. Although both studies had a large sample size, the recruitment of apparently healthy dogs could explain the lower detection rates. In our study, among 53 positives, 17 dogs (13 with both kidney and liver disease, 2 with kidney disease, 2 with liver disease) were in the leptospiremia phase, given that *Leptospira* spp. were detected only from whole blood; 11 dogs (6 with both kidney and liver disease, 5 with kidney disease) were in the leptospiruric phase, with positive detection only from urine samples. In another 25 dogs, *Leptospira* spp. were detected in both whole blood and urine samples, suggesting these dogs were in the period of active infection and actively shedding.

In this study, 10 leptospiral isolates were successfully recovered from eight dogs. One of the dogs had kidney disease and the other seven dogs were diagnosed with both kidney and liver disease (*n* = 7). The isolated strains were identified as *L. interrogans* serovar Bataviae (*n* = 8), *L. interrogans* serovar Australis (*n* = 2), and *L. borgpetersenii* serovar Javanica (*n* = 1). Serovar Bataviae has been reported in dogs as a primary reservoir or incidental host [36]. However, both Australis and Javanica are not adapted serovars for dogs [36]. In Malaysia, serovars Bataviae, Australis, and Javanica were reported as circulating serovars among rats [37,38]. This may suggest that the dogs diagnosed with leptospirosis could have been infected through environmental exposure, most likely acquired through contact with water or soil contaminated with leptospires shed in the urine of rats [1]. A direct link could not be proven in this study as the trapping of rats and soil sampling from the environment where the infected dogs lived were not possible. In this study, the laboratory diagnosis for dogs clinically suspected with leptospirosis employed PCR and if successful, culturing for isolation and identification was carried out. Nonetheless, there is the possibility that PCR or culture-negative dogs were actually infected with *Leptospira* spp. The confirmation of negativity by MAT using paired serum samples would have been ideal. 

Understanding the risk factors that affect the incidence of leptospirosis in any region will help increase a veterinarian’s index of suspicion about canine patients presenting with clinical signs and laboratory analysis consistent with leptospirosis. Previous reports on risk factors for canine leptospirosis have resulted in varying conclusions. In this study it was observed that medium and large breed dogs, especially with outdoor access and rat exposure, were more likely to be diagnosed with leptospirosis than small breed dogs, indoor dogs, and those without rat exposure, if presented with kidney and/or liver disease [39,40]. Large and medium breed dogs were at greater risk presumably because these dogs were maintained within the house compound and, thus, may have a higher likelihood of being exposed to leptospires and carriers such as rats. 

Previous studies have identified male dogs and older dogs as being at greater risk of leptospirosis [19,40,41,42,43,44,45]. However, in this study, sex and age did not appear as an identifiable risk factor for canine leptospirosis, as similarly reported by Meeyam et al., (2006) [46]. Having contact with infected dogs in a multidog household did not significantly increase the probability of infection [47], similarly multiple-dog households (*n* = 22) were not a significant risk factor. Healthy dogs were likely protected from infection even though they stayed together with infected dogs, because owners claimed that dogs were managed or kept separately, or the different behavioral factors (i.e., hunting, chasing, territorial) and/or immunity level of each dog. 

A history of rat exposure and acute clinical illness were important predictors of leptospirosis in dogs diagnosed with kidney and/or liver disease. Presence of rats has been shown to be significant predictor of dog seropositivity [11]. Early identification of these predictors from the history during consultation and with the corresponding laboratory results may alert the veterinarian, prioritizing leptospirosis as one of the differential diagnoses. Without this important information, it remains impossible to differentiate between infected or noninfected dogs purely by comparison of the kidney and liver functions. 

Human leptospirosis was reported as endemic in Malaysia [48]. However, leptospirosis in domestic and wild animals in Malaysia is often reported to occur sporadically across a wide range of serovars [49]. Although endemicity might be different between humans and animals, if the disease presents with a severe form, regardless of in humans or animals, it will lead to increased morbidity and mortality [2]. It is believed that early treatment intervention can be initiated to improve outcome for each infected dog. In this study, 35 out of 53 infected dogs survived with treatment. However, 18 out of 53 (34.0%) infected dogs did not survive, despite treatment, indicating the mortality rate was high. Therefore, it is of the utmost importance for veterinarians to identify the predictors for canine leptospirosis, so that clinicians can recognize the potentially fatal cases earlier rather than later, and carefully plan their treatment to support dogs with kidney and/or liver disease.

Leptospirosis can be treated with antibiotics such as doxycycline, but at times, the disease will progress from an acute phase, in which severe clinical signs appear with a sudden onset, to a chronic phase, in which the infection persists but progresses less aggressively [15,50]. This study showed that infected dogs that received antibiotics at an early stage of the disease (prior to presentation at the hospital or clinic) and dogs with chronic clinical illness, both had a higher chance of survival post-infection compared to the dogs that were not treated with antibiotics prior to presentation and those with acute clinical illness. Eight infected dogs that presented with chronic clinical illness that did not receive antibiotics (prior to presentation at the hospital or clinic) survived the infection during hospitalization in this study. Perhaps, anti-leptospiral antibodies might have developed in these dogs, assisting their survival. 

Dogs vaccinated annually remained at risk, as vaccination does not provide cross-protection against non-vaccinal serovars [51]; three out of eight dogs with positive isolates in this study were vaccinated. This is a reminder of the limitations of the existing vaccines. The isolation of serovars Bataviae, Australis, and Javanica in this study was alarming, due to their absence in commercial vaccines, and these serovars might be circulating among the local dog population. Among the eight dogs with positive isolates, seven dogs presented with acute clinical illness where *L. interrogans* serovars Bataviae and Australis were isolated, whilst one dog (D63) presented with chronic clinical illness where *L. borgpetersenii* serovar Javanica was isolated. This could suggest that leptospires can be isolated from either the acute or chronic phases of infection. Therefore, proper characterization of leptospiral isolates remains a crucial bottleneck to assess the role of particular serovars or strains in the epidemiology of canine leptospirosis and may provide evidence-based knowledge to support the development and commercialization of multivalent vaccines containing serovars that are circulating among local populations.

## 5. Conclusions

This study showed that approximately one in three dogs presenting with kidney and/or liver disease in Selangor Malaysia was infected with leptospirosis. Knowing the relevant factors (rat exposure, medium and large breed dogs, the association with outdoor activities and acute clinical illness) that can be obtained from the initial evaluation (patient signalment and history taking) and that are predictive of, or elevate the risk of, canine leptospirosis, will help veterinarians identify at-risk dogs, especially dogs presenting with kidney and/or liver disease, and initiate appropriate treatment for the dogs. Veterinarians can advise on the prevention and control of leptospirosis for pet owners, such as using rodent pest control, practicing good hygiene, and wearing gloves when handling infected dogs, given that leptospirosis is a zoonotic disease with a high mortality rate if the disease is not diagnosed and treated as early as possible.

## Figures and Tables

**Table 1:** The average and standard deviation of the kidney and liver profile in dogs diagnosed with kidney and/or liver disease, and with and without leptospirosis, of those recruited. Leptospirosis was confirmed by PCR.

Positive Leptospirosis (*n* = 53)	Negative Leptospirosis (*n* = 71)
Urea: 46.7 ± 51.3 mmol/L Creatinine: 326.2 ± 458.0 µmol/L	Urea: 34.0 ± 24.1 mmol/L Creatinine: 343.1 ± 339.6 µmol/L
ALT: 154.9 ± 138.6 U/L ALP: 370.5 ± 753.3 U/L	ALT: 179.6 ± 534.5 U/L ALP: 314.7 ± 400.4 U/L

Note: biochemical parameter: average ± SD (standard deviation); mmol/L is millimoles per liter; µmol/L is micromoles per liter; U/L is units per liter; ALT is alanine aminotransferase; ALP is alkaline phosphatase.

**Table 2:** Demographic data of the dogs diagnosed with kidney and/or liver disease (*n* = 124) presented as number of dogs and percentage (%).

Demographic	No. of Dogs (%)	Demographic	No. of Dogs (%)
Age (years old) * Young (≤1) Adult (<1–6) Senior (≥6)	13 (10.5%) 48 (38.7%) 63 (50.8%)	Management Indoor Outdoor	51 (41.1%) 73 (58.9%)
Breed * Large Medium Small	31 (22.6%) 65 (52.4%) 28 (25.0%)	Type of household Single Multiple	68 (54.8%) 56 (45.2%)
Sex Male Female	76 (61.3%) 48 (38.7%)	Rat exposure Exposed Not exposed	78 (62.9%) 46 (37.1%)
Vaccination status Vaccinated Non-vaccinated	52 (41.9%) 72 (58.1%)	Clinical illness (days) Acute (≤7) Chronic (>7)	102 (82.3%) 22 (17.7%)

Note: ≤ is less than or equal to; < is less than; ≥ is more than or equal to; > is more than. * Age and breed were recorded based on published guidelines [25].

**Table 3:** The serotyping and MLST results of *Leptospira* spp. isolates (*n* = 10) recovered from specific dogs and the type of samples (*n* = 8).

Dog ID	Sample Obtained	Identification	
		Serotyping	MLST
D2	Urine	Bataviae	ST 50—*L. interrogans* serogroup Bataviae
D19	Blood	Bataviae	ST 50—*L. interrogans* serogroup Bataviae
	Urine	Bataviae	ST 50—*L. interrogans* serogroup Bataviae
D27	Urine	Bataviae	ST 50—*L. interrogans* serogroup Bataviae
D41	Urine	Bataviae	ST 50—*L. interrogans* serogroup Bataviae
D52	Urine	Bataviae	ST 50—*L. interrogans* serogroup Bataviae
D63 *	Blood	Javanica	ST 143—*L. borgpetersenii* serogroup Javanica
D82 *	Urine	Australis	ST 51—*L. interrogans* serogroup Australis
	Blood	Australis	ST 51—*L. interrogans* serogroup Australis
D85 *	Urine	Bataviae	ST 50—*L. interrogans* serogroup Bataviae

Note: * Dogs had been vaccinated annually with a commercial tetravalent vaccine (serovars Canicola, Grippotyphosa, Icterohaemorrhagiae, and Pomona); MLST is multilocus sequence typing; ST is sequence type.

**Table 4:** Eight risk factors associated with leptospiral infection in dogs diagnosed with kidney and/or liver disease (*n* = 124) based on univariate analyses.

Factors	Pearson Chi-Square	*p*-Value	Odds Ratio	95% CI
Age				
Adult/Young	0.013	1.000	1.07	0.31–3.67
Senior/Young	0.293	0.757	0.72	0.22–2.39
Senior/Adult	1.076	0.336	0.67	0.31–1.43
Breed				
Medium/Small *	7.342	0.010	4.19	1.42–12.38
Large/Small *	8.604	0.006	5.59	1.69–18.51
Large/Medium	0.429	0.663	1.33	0.56–3.14
Sex				
Male/Female	0.306	0.710	0.81	0.39–1.69
Vaccination status				
Vaccinated/Not vaccinated	0.203	0.715	0.85	0.41–1.75
Management				
Outdoor/Indoor	0.087	0.854	1.12	0.54–2.30
Type of household				
Multiple/Single	0.498	0.585	0.77	0.38–1.58
Rat exposure				
Exposed/Not exposed *	8.289	0.005	3.14	1.42–6.95
Clinical illness				
Acute/Chronic	2.921	0.087	0.45	0.18–1.14

Note: * significant at *p*-value < 0.05; 95% CI is 95% confidence interval of lower limit and upper limit.

**Table 5:** Pearson chi-square analysis for rat exposure, based on type of management (*n* = 53).

Factors	Pearson Chi-Square	*p*-Value	Odds Ratio	95% CI
Type of management Outdoor/Indoor *	4.742	0.045	4.31	1.10–16.93

Note: * significant at *p*-value < 0.05; 95% CI is 95% confidence interval of lower limit and upper limit.

**Table 6:** Six predictive factors affecting leptospiral positivity in dogs diagnosed with kidney and/or liver disease (*n* = 124), based on multivariate logistic regression.

Factors	Simple Logistic Regression			Multiple Logistic Regression ^a^		
	b	Crude OR (95% CI)	*p*-Value	b	Adjusted OR (95% CI)	*p*-Value
Sex (Male/Female)	−0.125	0.88 (0.40, 1.95)	0.757			
Vaccination (Vaccinated/Not vaccinated)	−0.133	0.88 (0.40, 1.92)	0.740			
Type of Management (Outdoor/Indoor)	−0.002	1.00 (0.45, 2.24)	0.996			
Household (Multiple/Single)	−0.455	0.64 (0.29, 1.39)	0.254			
Rat exposure (Exposed/Not exposed)	1.347	3.85 (1.60, 9.23)	0.003	1.258	3.52 (1.54, 8.03)	0.003
Clinical illness (Acute/Chronic)	1.002	2.72 (0.99, 7.52)	0.053	1.015	2.76 (1.01, 7.51)	0.047

Note: ^a^ backward likelihood ratio (LR) multivariate multiple logistic regression was applied. Multicollinearity and interaction were checked. A Hosmer–Lemeshow test (*p* > 0.05), classification table (overall correctly classified percentage = 63.7%) and area under the ROC curve (0.71) were applied to check the model fitness, r^2^ = 0.13; 95% CI is 95% confidence interval of lower limit and upper limit, b is unstandardized beta.

**Table 7:** Association between antibiotic given prior presentation, duration of clinical illness and vaccination status with the survival of the dogs using Pearson Chi-Square analysis (*n* = 53) in univariate analysis.

Factors	Pearson Chi-Square	*p*-Value	Odds Ratio	95% CI
Antibiotic				
Given/None given	5.150	0.036	4.72	1.16–19.26
Clinical illness				
Chronic/Acute *	5.300	0.040	8.87	1.05–74.95
Vaccination				
Vaccinated/Not vaccinated	0.006	1.000	1.05	0.33–3.36

Note: * significant at *p*-value < 0.05; 95% CI is 95% confidence interval of lower limit and upper limit.

## Data Availability

The data that support the findings of this study are available from the corresponding author upon request.
